# Salvage microsurgery for vestibular schwannoma after failed stereotactic radiosurgery: a multi-institutional retrospective study

**DOI:** 10.1007/s11060-026-05598-0

**Published:** 2026-05-20

**Authors:** Filippo Friso, Noa Ben Dor, Alfredo Conti, Alfredo Decio Fabbri, Federico Calvaruso, Virginia Dallari, Francesca De Cecco, Franco Federico Bochicchio, Emanuele La Corte, Tomasz Wojciechowski, Kazimierz Niemczyk, Adrian Drożdż, Grzegorz Turek, Lukas Anschuetz, Davide Soloperto, Giacomo Pavesi, Barbara Masotto, Daniele Marchioni, Carmelo Sturiale, Paolo Ferroli, Francesco DiMeco, Livio Presutti, Giulia Molinari

**Affiliations:** 1 Department of Neurosurgery, IRCCS Institute of Neurological Sciences of Bologna, Bologna, Italy; 2 Department of Medical and Surgical Sciences, Alma Mater Studiorum University of Bologna, Bologna, Italy; 3 Department of Otorhinolaryngology-Head and Neck Surgery, IRCCS University Hospital of Bologna, Bologna, Italy; 4 Department of Otorhinolaryngology-Head and Neck Surgery, University Hospital of Modena, Modena, Italy; 5 Department of Otorhinolaryngology-Head and Neck Surgery, University of Verona, Verona, Italy; 6 Department of Health Sciences, Università degli Studi di Milano, Milan, Italy; 7 Department of Neurosurgery, Fondazione IRCCS Istituto Neurologico “Carlo Besta”, Milan, Italy; 8 Neurosurgery and Neurotraumatology Unit, Department of Neurosciences, IRCCS Ospedale Policlinico San Martino, Genoa, Italy; 9 Department of Otorhinolaryngology, Head and Neck Surgery, Medical University of Warsaw, Banacha 1a St, Warsaw, 02-097 Poland; 10 Department of Neurosurgery, Brodno Masovian Hospital, Kondratowicza 8 St, Warsaw, 03-242 Poland; 11 Department of Otorhinolaryngology, Head and Neck Surgery, CHUV, University of Lausanne, Lausanne, Switzerland; 12 The Sense Innovation and Research Center, Lausanne, Sion, Lausanne, Switzerland; 13 Department of Neurosurgery, University of Modena and Reggio Emilia, Modena, Italy; 14 Department of Neurosurgery, University Hospital of Verona, Verona, Italy; 15 Department of Pathophysiology and Transplantation, University of Milan, Milan, Italy; 16 Department of Neurosurgery, Johns Hopkins Medical School, Baltimore, MD USA

**Keywords:** Vestibular schwannoma, Acoustic neuroma, Recurrence, Stereotactic radiosurgery, Radiation therapy, Salvage microsurgery, Oncology

## Abstract

**Objective:**

The management of sporadic vestibular schwannoma (VS) with stereotactic radiosurgery (SRS) is becoming increasingly common worldwide. Despite its efficacy, treatment failure can occur in a subset of patients. This study aimed to describe the clinical outcomes of salvage microsurgery following failed primary SRS in patients with sporadic VS.

**Methods:**

This retrospective study included adult patients (≥ 18 years) who underwent salvage microsurgery following failed primary SRS at six European tertiary referral centers. Data collection was performed from January 2012 to December 2022, and data analysis was performed from July to September 2025.

**Results:**

Among 28 patients (15 men, 13 women), surgery indication was radiological regrowth in 27/28 patients. The median interval from SRS to salvage microsurgery was 48 months (range, 24–120), and the median age at time of salvage microsurgery was 53 years (range, 30–74). Axial diameter increased from median 18.5 mm (range, 7.5–27) before SRS to 25 mm (range, 10.5–33) before surgery. Gross total resection was achieved in 13/28 (46.4%). Complications occurred in 3/28 patients (10.7%). Serviceable hearing (AAO-HNS class A/B) declined from 10/20 patients (50.0%) pre-SRS to 4/24 (16.7%) preoperatively; all patients with postoperative follow-up (14/28) were AAO-HNS class D. Preoperative facial nerve function was House–Brackmann (HB) grade I in 25/28 patients (89.3%); good facial function (HB I–II) was seen in 14/27 (51.9%) at discharge and 11/15 (73.3%) at 12 months, with 3/15 (20.0%) remaining HB grade V–VI.

**Conclusion:**

Salvage microsurgery is a viable therapeutic option for managing VS after failed SRS. GTR or NTR with a relatively low complication profile is achievable, although hearing preservation is not a realistic goal in this setting, whereas facial nerve function may improve postoperatively.

## Introduction

Vestibular schwannomas (VS) are benign tumors that arise from Schwann cells in the vestibular division of the eighth cranial nerve. These lesions are the most prevalent neoplasm of the cerebellopontine angle (CPA) and typically present with progressive unilateral hearing loss, tinnitus, and imbalance, and less commonly with facial weakness or trigeminal dysfunction [1–4]. The widespread application of high-resolution MRI has contributed to an increased detection rate of small, often asymptomatic tumors, particularly in older adults [3, 5].

Historically, microsurgical resection was the primary treatment modality; however, the therapeutic landscape has evolved markedly with the advent and refinement of stereotactic radiosurgery (SRS) [6–8]. SRS is now widely accepted as a first-line intervention for small- to medium-sized VS, typically those measuring ≤ 2.5 to 3.0 cm in maximal extracanalicular diameter, owing to its high long-term tumor control rates (91–98% at five years) and favorable safety profile in appropriately selected patients [5, 9].

Despite its high efficacy, treatment failure following SRS occurs in a small proportion of patients, with reported rates ranging from 2.5% to 5%. Contributing factors may include suboptimal marginal dosing, inadequate isocenter coverage, and intrinsic tumor radioresistance [5, 9–16]. In such cases, salvage management options include repeat radiosurgery or microsurgical resection, both of which carry an elevated risk of morbidity due to radiation-induced tissue alterations [5, 9, 11, 16–21].

Salvage microsurgical resection is particularly demanding because prior irradiation frequently obscures normal anatomical landmarks and increases the technical complexity of tumor dissection. Consequently, these procedures require substantial surgical expertise, advanced intraoperative neurophysiological monitoring, and carefully individualized treatment planning to optimize functional outcomes [5, 9, 16, 17, 19, 22].

Given the rarity of these cases, a European multi-institutional collaboration was initiated with the primary objective of characterizing the indications, operative challenges, and postoperative outcomes among patients with sporadic vestibular schwannoma who underwent salvage microsurgery following failed primary SRS.

## Materials and methods

### Study design and setting

This multi-institutional retrospective study was conducted at six European tertiary referral centers in Bologna (Italy), Modena (Italy), Verona (Italy), Milan (Italy), Warsaw (Poland), and Bern (Switzerland). The study period spanned January 2012 to December 2022; the end date was chosen to allow for at least 12 months of clinical follow-up for functional outcomes in a subset of patients. Each center performed a systematic query of operative logs and institutional VS/radiosurgery databases to identify consecutive patients who underwent salvage microsurgical resection after primary SRS treatment. Case lists were reviewed locally by the skull base teams to maximize the completeness of case ascertainment.

### Eligibility criteria

Eligible participants included adult patients (≥ 18 years) with unilateral sporadic VS who received SRS as the initial definitive treatment and subsequently underwent salvage microsurgical resection. We defined SRS failure as either (1) progressive radiological tumor growth, characterized as a > 20% increase in tumor volume compared with the earliest post-SRS MRI, confirmed on serial imaging and occurring outside the expected post-SRS pseudoprogression window, or (2) clinically significant neurological deterioration attributable to the tumor (e.g., trigeminal neuralgia, facial dysfunction, hydrocephalus) prompting surgical management, even in the absence of a clear volumetric increase [23–25]. Patients were excluded if they had undergone microsurgical resection prior to SRS, had a diagnosis of neurofibromatosis type 2 (NF2), showed malignant transformation of the tumor post-radiosurgery, or were treated with cerebrospinal fluid diversion alone without tumor removal.

### Clinical data

Clinical, audiometric, radiological, and surgical data were retrospectively extracted from institutional databases, medical records, and intraoperative video reviews. The demographic variables included age and sex. Tumor characteristics included the Koos classification (pre-SRS and pre-surgery) [26], internal auditory canal (IAC) involvement, and quantitative tumor size when available (maximum extracanalicular axial diameter measured parallel to the petrous ridge).

Hearing status was recorded at three time points (pre-SRS, pre-surgery, and post-surgery when available) using pure-tone audiometry and the American Academy of Otolaryngology–Head and Neck Surgery (AAO-HNS) classification [27–29]. Facial nerve function was assessed preoperatively, at discharge, and at 3, 6, and 12 months (when available) using the House–Brackmann (HB) grading system [29, 30].

The radiosurgical variables included the device type (Gamma Knife, CyberKnife, or LINAC-based), number of sessions, and delivered dose when available. Surgical variables included the surgical approach, intraoperative findings, and extent of resection (EOR). Residual tumor was assessed using volumetric comparison between preoperative and first postoperative contrast-enhanced T1-weighted MRI (typically obtained 2–3 months after surgery). EOR was classified as gross total resection (GTR; no radiographic residual), near-total resection (NTR; residual volume ≤ 2%), subtotal resection (STR; residual volume > 2% and < 5%), and partial resection (PR; residual volume ≥ 5%) [31]. We acknowledge that proportional thresholds do not account for absolute residual size; volumetrically defined absolute thresholds for NTR/STR have been proposed recently [32] but could not be applied uniformly in this retrospective multicenter cohort due to heterogeneous imaging workflows and incomplete availability of standardized 3D volumetry outputs across the centers.

Beyond the early postoperative MRI used for residual assessment, subsequent radiological follow-up was heterogeneous and incompletely documented; therefore, time-specific tumor control rates after salvage surgery could not be calculated. Importantly, owing to the rarity of salvage cases and the multi-institutional retrospective design, missing data were expected for some variables; therefore, all denominators are reported explicitly in the Results section.

## Results

A total of 28 patients met the inclusion criteria, comprising 15 men and 13 women. Cases were contributed by Bologna (*n* = 1), Modena (*n* = 1), Verona (*n* = 10), Milan (*n* = 10), Warsaw (*n* = 5), and Bern (*n* = 1). The demographic, tumor, radiosurgery characteristics, and surgical outcomes of the study population are summarized in Table 1.

### Tumor characteristics

Quantitative tumor size data were available for a subset of the patients. The maximum extra canalicular axial diameter (measured parallel to the petrous ridge) prior to radiosurgery was available in 16/28 patients, with a median of 19 mm (range 7.5–27). The same measurement prior to salvage surgery was available in 21/28 patients, with a median of 24.4 mm (range, 10.5–33). Among patients with paired measurements (*n* = 16), the axial diameter increased from radiosurgery to the time of surgery.

**Fig. 1:**
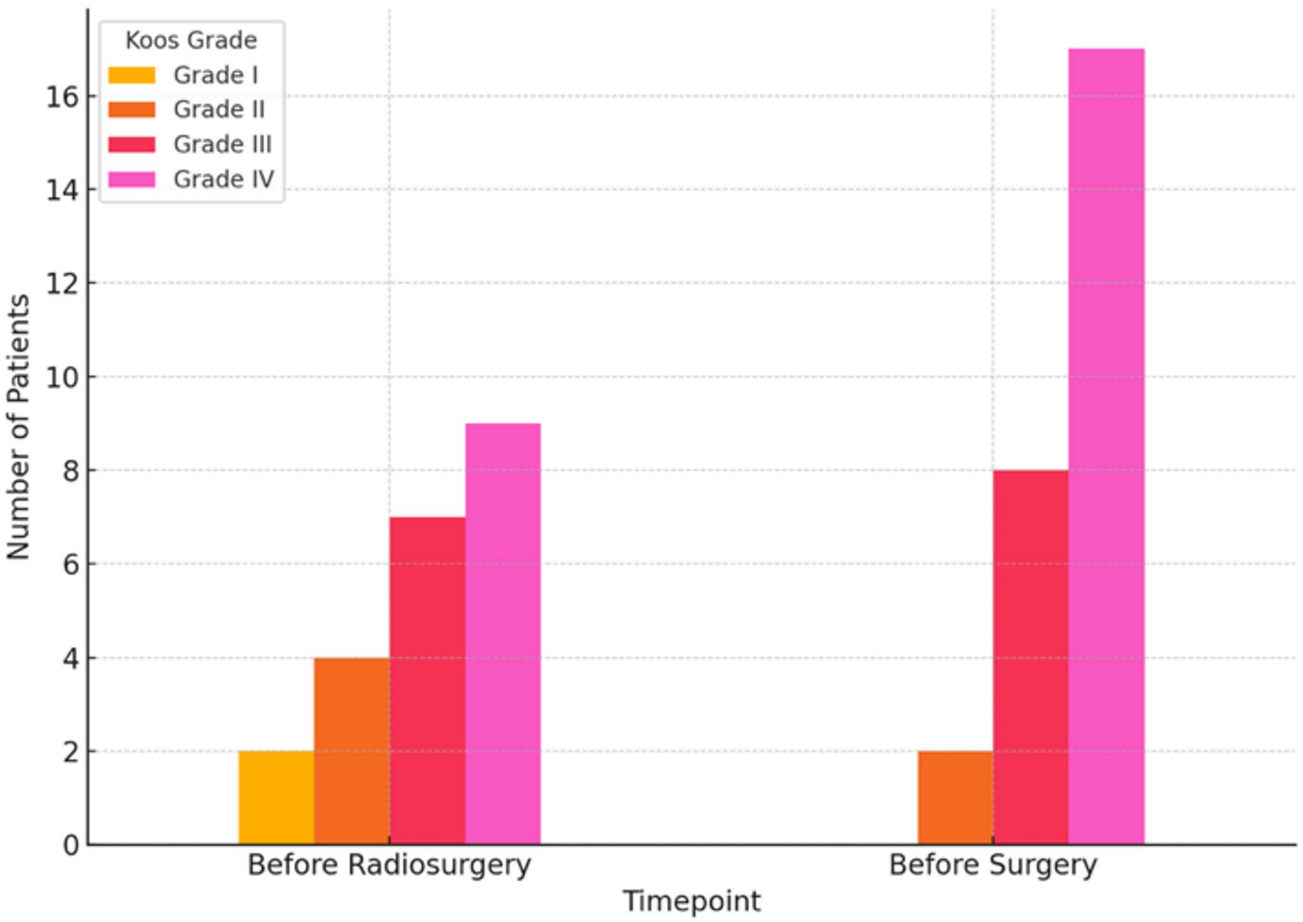
Bar chart showing the number of patients according to the Koos classification at two time points: before radiosurgery and before surgical intervention

The Koos classification was documented at the time of stereotactic radiosurgery and prior to salvage surgery in 22 and 27 patients, respectively (Fig. 1). Before radiosurgery, Koos grades I–IV were present in two (9.1%), four (18.2%), seven (31.8%), and nine (40.9%) patients, respectively. At the time of salvage surgery, a higher proportion of advanced tumors was observed, with two patients (7.4%) classified as grade II, eight (29.6%) as grade III, and 17 (63.0%) as grade IV (Koos data missing in one patient at the surgical time point).

### Radiosurgery and microsurgery characteristics

The most commonly used modality was the Gamma Knife (20/28, 71.4%), followed by CyberKnife (6/28, 21.4%) and LINAC-based SRS (2/28, 7.1%). The number of sessions and dose information were incompletely documented and thus not reported.

The median interval between radiosurgery and salvage microsurgery was 48 months (range, 24–120 months), corresponding to 4 years (range, 2–10 years). The most frequent indication for surgery was radiological tumor regrowth without new or progressive symptoms (15/28, 53.6%). Tumor regrowth associated with clinically relevant symptoms (e.g., trigeminal neuralgia, hemifacial spasm, and hydrocephalus) occurred in 12/28 patients (42.9%). One patient (3.6%) underwent surgery due to symptomatic worsening in the absence of measurable tumor growth.

Microsurgical resection was performed via the retrosigmoid approach in 25/28 patients (89.3%), translabyrinthine approach in 2/28 (7.1%), and transpromontorial approach in 1/28 (3.6%). Intraoperative findings revealed firm, poorly mobile tumors due to arachnoid adhesions or post-radiation changes in 19/28 cases (67.9%). Gross total resection was achieved in 13/28 patients (46.4%), near-total resection (≤ 2% residual) in 6/28 (21.4%), subtotal resection (> 2% and < 5% residual) in 8/28 (28.6%), and partial resection (≥ 5% residual) in 1/28 (3.6%). Postoperative complications were observed in three patients (10.7%). These included one case of meningitis, one case of cerebrospinal fluid leakage, and one case of atrial fibrillation.

### Clinical outcomes

Pre-SRS hearing class (AAO-HNS) was available in 20/28 patients: 7 were class A, 3 were class B, 5 were class C, and 5 were class D. Preoperative (pre-microsurgery) hearing class was available in 24/28 patients: 2 were class A, 2 were class B, 8 were class C, and 12 were class D. Among the 14 patients with audiometric follow-up available after salvage surgery (50%), all were classified as AAO-HNS Class D, indicating non-serviceable hearing.

**Fig. 2:**
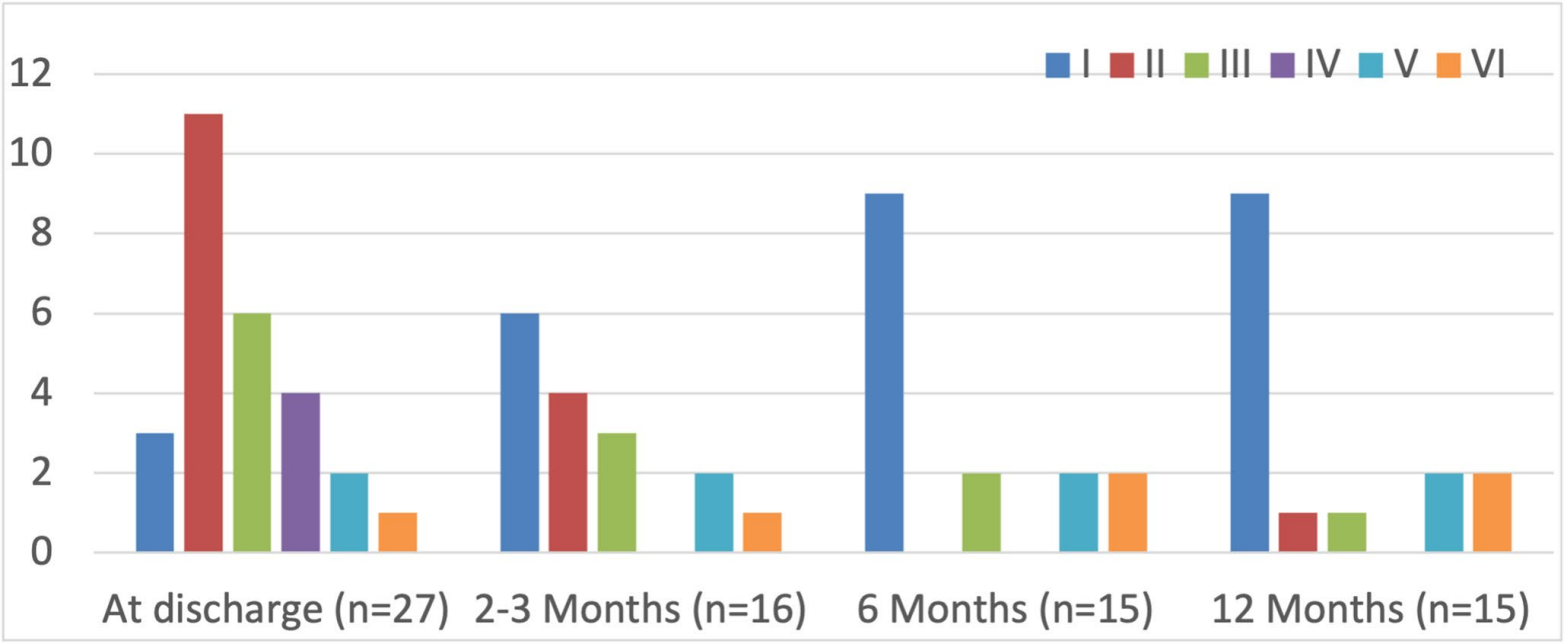
Progression of facial nerve function following salvage microsurgery for vestibular schwannoma (house-brackmann grades I–VI)

The median duration of facial nerve follow-up was 64 months (range, 3–120 months). Preoperative facial nerve function was HB grade I in 25/28 patients and HB grade II in 3/28 patients (including two patients with hemifacial spasm). At discharge, facial nerve outcome was evaluated in 27 out of 28 patients, with HB grades I–VI observed in three, eleven, six, four, two, and one patient, respectively. At the 12-month follow-up (*n* = 15), 10 patients had recovered to HB grade I, one to grade II, one to grade III, and three remained in HB grades five to six. These trends are illustrated in Fig. 2 and are detailed in Table 2.

**Table 1:** Baseline characteristics, radiosurgery parameters, and surgical outcomes (*N*= 28)

Characteristic	Subgroup	No. (%) / median (range)
Sex	Male	15 (53.6%)
	Female	13 (46.4%)
Age at radiosurgery, median (range), y		48 (24–70)
Primary SRS modality	Gamma Knife	20 (71.4%)
	CyberKnife	6 (21.4%)
	LINAC	2 (7.1%)
Interval from SRS to salvage surgery, median (range), months		48 (24–120)
Reason for surgical indication	Regrowth without symptoms	15 (53.6%)
	Regrowth with symptoms	12 (42.9%)
	Worsening symptoms without growth	1 (3.6%)
Koos grade prior to salvage surgery (*n* = 27)	Grade II	2 (7.4%)
	Grade III	8 (29.6%)
	Grade IV	17 (63.0%)
Axial tumor diameter prior to radiosurgery (*n* = 16), median (range), mm		19 (7.5–27)
Axial tumor diameter prior to salvage surgery (*n* = 21), median (range), mm		24.4 (10.5–33)
Serviceable hearing (AAO-HNS class A/B) prior to radiosurgery (*n* = 20)		10/20 (50.0%)
Serviceable hearing (AAO-HNS class A/B) prior to salvage surgery (*n* = 24)		4/24 (16.7%)
Serviceable hearing (AAO-HNS class A/B) after salvage surgery (*n* = 14)		0/14 (0.0%)
Facial nerve function prior to salvage surgery	HB grade I	25 (89.3%)
	HB grade II	3 (10.7%)
Surgical approach	Retrosigmoid	25 (89.3%)
	Translabyrinthine	2 (7.1%)
	Transpromontorial	1 (3.6%)
Intraoperative adhesions/fibrosis	Present	19 (67.9%)
	Absent/Not described	9 (32.1%)
Extent of resection	Gross total (GTR)	13 (46.4%)
	Near-total (≤ 2%)	6 (21.4%)
	Subtotal (> 2–<5%)	8 (28.6%)
	Partial (≥ 5%)	1 (3.6%)
Postoperative complications	Any complication	3 (10.7%)
	Meningitis	1 (3.6%)
	CSF leak	1 (3.6%)
	Atrial fibrillation	1 (3.6%)

**Table 2:** Facial nerve function (House–Brackmann grade) over time. Note: N indicates the number of patients with documented HB grades at each time point; follow-up completeness varied across institutions

Time point	*N*	HB I	HB II	HB III	HB IV	HB V	HB VI
Before microsurgery	28	25	3	0	0	0	0
Discharge	27	3	11	6	4	2	1
3 months	16	6	4	3	0	2	1
6 months	15	9	0	2	0	2	2
12 months	15	10	1	1	0	2	1

## Discussion

This multi-institutional retrospective study contributes to the expanding body of literature on the outcomes of salvage microsurgery for VS after failed SRS. As SRS continues to be widely adopted as a primary management strategy for small- to medium-sized VS, the frequency of salvage surgical interventions, although still relatively uncommon, may increase over time. In this evolving landscape, a deeper understanding of the indications, technical challenges, and outcomes of salvage procedures warrants further investigation.

### Indications and timing for salvage intervention

In our cohort, radiological tumor regrowth was the predominant indication for salvage microsurgery (27/28, 96.4%). These findings are in concordance with the most recent systematic review and meta-analysis by Ribeiro et al. [5] and are further supported by earlier studies [9, 19, 33, 34]. Standardized definitions of radiological failure are essential when interpreting tumor control and failure rates in the post-SRS setting, particularly when cohorts are restricted to actively growing tumors [22].

The median interval between SRS and surgical intervention was 48 months, reflecting the clinical preference for delayed surgery in the absence of acute neurological deterioration [5, 9, 35, 36]. This strategy also facilitates the differentiation between true progression and post-SRS pseudoprogression, a transient swelling that typically occurs within the first 6–18 months after treatment [37–39]. Crucially, surgical intervention during this period is associated with increased operative risk owing to obscured anatomical planes and the heightened vulnerability of adjacent neurovascular structures. Therefore, delayed surgery, ideally beyond 24 to 36 months, has become the preferred strategy for patients without urgent clinical deterioration [10, 36–38, 40–42].

### Considerations on pre-radiosurgery tumor volume

A noteworthy characteristic of our salvage cohort was the substantial tumor burden at the time of initial SRS. Among the 22 patients with available pre-SRS Koos data, 16 (72.7%) were classified as Koos grade III–IV at the time of radiosurgery. In patients with quantitative size data, the median axial diameter prior to SRS was 19 mm and increased to 24.4 mm prior to salvage surgery, with enlargement observed in all patients with paired measurements. Although this study was not designed to identify predictors of SRS failure, the predominance of higher Koos grades and increasing tumor size in this cohort underscores the importance of careful patient selection and counseling when considering primary SRS for larger tumors, where the balance between tumor control and functional preservation may be less favorable [22, 25, 43].

### Operative challenges

The intraoperative findings in our cohort highlight the inherent complexity of salvage microsurgery in previously irradiated VS. Radiation-induced alterations, most notably fibrosis and arachnoid adhesions, were encountered in 67.9% of cases, and several tumors initially graded as Koos II demonstrated surgical characteristics typical of grade IV lesions, including dense adhesions to the brainstem and facial nerve, loss of arachnoidal planes, and friable tumor consistency. These findings suggest that in the salvage setting, the Koos classification, which has traditionally served as a radiological staging tool and guide for treatment selection [26], may not reliably reflect the surgical complexity posed by radiation-induced anatomical distortion [36, 44], as it is associated with obscure surgical planes, increases the difficulty of internal debulking and extracapsular dissection, and heightens the risk of cranial nerve injury [15, 18, 19, 39, 40, 44–48].

The clinical implications are apparent in the degree of resection, with gross total resection (GTR) accomplished in fewer than half of the cases. This is in line with recent multicenter data showing a higher likelihood of foregoing GTR during salvage surgery than during primary microsurgery, particularly as tumor size increases [34]. From a functional preservation perspective, recent volumetric studies suggest that leaving a very small residual tumor (NTR) may offer a “win–win” balance between long-term tumor control and facial nerve preservation [2, 5, 9, 11, 12, 32, 46, 49].

However, in our study, radiological follow-up beyond early postoperative MRI used to assess residual disease was heterogeneous and incompletely captured across centers, precluding robust time-to-event analyses of tumor regrowth after salvage surgery. Future collaborative studies with standardized volumetry and predefined imaging intervals are needed to better define the oncological durability after salvage resection.

Notably, our complication rate was relatively low (10.7%) and included only one case each of meningitis, cerebrospinal fluid leakage, and atrial fibrillation. This compares favorably with published reports [5, 15, 17, 19] and likely reflects careful patient selection and experience at high-volume centers. Importantly, no procedure-related mortality occurred, consistent with the experience of other high-volume centers and underscoring the safety of salvage MS when performed by experienced teams with appropriate patient-selection criteria [11, 46].

### Functional outcomes

Hearing preservation was not achieved in any of the patients in our cohort, including the four patients who had serviceable hearing (AAO-HNS class A/B) before salvage surgery and deteriorated postoperatively to AAO-HNS class D. This outcome might be explained by cumulative cochlear and neural injury from tumor growth, radiation, and surgery [13, 23, 24, 46, 50]. In this context, we argue that hearing preservation should not be considered a realistic goal of salvage surgery.

Long-term facial nerve follow-up beyond 12 months was available for 14 patients (median, 64 months). Facial nerve outcomes have improved over time. At discharge, 14/27 patients (51.9%) had good facial function (HB grade I–II), which improved to 11/15 patients (73.3%) at 12 months among those with available follow-up. Nonetheless, a clinically relevant subset (3/15, 20.0%) remained in HB grades V–VI at 12 months, underscoring the importance of preoperative counseling and a function-preserving operative strategy in this challenging population [14, 33, 39].

## Limitations

There are notable limitations to the current study, inherent to its retrospective multi-institutional design and the relative rarity of salvage microsurgery after failed SRS. The small sample size precluded inferential statistics or robust risk factor analyses. Baseline variables (audiometry, quantitative size/volumetry, radiosurgical dosimetry) and follow-up were inconsistently captured across institutions; therefore, denominators varied by endpoint/time point, and interval-specific tumor control after salvage surgery could not be calculated. Outcomes were derived from routine clinical documentation rather than from standardized assessments; therefore, incomplete case ascertainment and selection/referral bias cannot be excluded. Because only patients undergoing salvage surgery were included and no control group was available, the study could not estimate overall SRS failure rates or compare salvage strategies. Finally, percentage-based EOR categories may not reflect the impact of small, absolute residuals. Prospective registries with predefined endpoints are needed, although they may prove challenging given the relative rarity of the studied events.

## Conclusions

Salvage microsurgery is a viable therapeutic option for managing vestibular schwannomas after failed stereotactic radiosurgery. Despite inherent challenges, such as radiation-induced fibrosis and altered surgical planes, experienced multidisciplinary teams can achieve acceptable rates of tumor resection with a relatively low complication profile. Hearing preservation is not a realistic goal in this setting, whereas facial nerve function may improve postoperatively, although a subset of patients may experience persistent deficits. Given the retrospective design, limited sample size, and incomplete long-term follow-up, these findings are descriptive and hypothesis-generating in nature. Prospective multicenter studies with standardized imaging and functional assessments are needed to better define oncological durability and optimize functional outcomes after salvage surgery.

## Data Availability

The dataset analyzed in the current study is available from the corresponding author upon reasonable request.
